# Mesenchymal Stem Cells Coated with Synthetic Bone-Targeting Polymers Enhance Osteoporotic Bone Fracture Regeneration

**DOI:** 10.3390/bioengineering7040125

**Published:** 2020-10-12

**Authors:** Yuliya Safarova (Yantsen), Farkhad Olzhayev, Bauyrzhan Umbayev, Andrey Tsoy, Gonzalo Hortelano, Tursonjan Tokay, Hironobu Murata, Alan Russell, Sholpan Askarova

**Affiliations:** 1 Laboratory of Bioengineering and Regenerative Medicine, National Laboratory Astana, Nazarbayev University, Nur-Sultan 010000, Kazakhstan; yantsen@nu.edu.kz (Y.S.(Y.)); folzhayev@nu.edu.kz (F.O.); bauyrzhan.umbayev@nu.edu.kz (B.U.); andrey.tsoy@nu.edu.kz (A.T.); 2 School of Engineering and Digital Sciences, Nazarbayev University, Nur-Sultan 010000, Kazakhstan; 3 School of Sciences and Humanities, Nazarbayev University, Nur-Sultan 010000, Kazakhstan; gonzalo.hortelano@nu.edu.kz (G.H.); tursonjan.tokay@nu.edu.kz (T.T.); 4 Department of Chemical Engineering, Carnegie Mellon University, Pittsburgh, PA 15213, USA; hiromura@andrew.cmu.edu (H.M.); alanrussell@cmu.edu (A.R.)

**Keywords:** osteoporosis, bone fracture, mesenchymal stem cells, targeted cell delivery

## Abstract

Osteoporosis is a progressive skeletal disease characterized by reduced bone density leading to bone fragility and an elevated risk of bone fractures. In osteoporotic conditions, decrease in bone density happens due to the augmented osteoclastic activity and the reduced number of osteoblast progenitor cells (mesenchymal stem cells, MSCs). We investigated a new method of cell therapy with membrane-engineered MSCs to restore the osteoblast progenitor pool and to inhibit osteoclastic activity in the fractured osteoporotic bones. The primary active sites of the polymer are the N-hydroxysuccinimide and bisphosphonate groups that allow the polymer to covalently bind to the MSCs’ plasma membrane, target hydroxyapatite molecules on the bone surface and inhibit osteolysis. The therapeutic utility of the membrane-engineered MSCs was investigated in female rats with induced estrogen-dependent osteoporosis and ulnar fractures. The analysis of the bone density dynamics showed a 27.4% and 21.5% increase in bone density at 4 and 24 weeks after the osteotomy of the ulna in animals that received four transplantations of polymer-modified MSCs. The results of the intravital observations were confirmed by the post-mortem analysis of histological slices of the fracture zones. Therefore, this combined approach that involves polymer and cell transplantation shows promise and warrants further bio-safety and clinical exploration.

## 1. Introduction

Osteoporosis is a chronic progressive metabolic bone disease that predisposes patients to an increased risk of bone fracture. Each year, there are more than 9 million fractures associated with osteoporosis, including 1.6 million hip fractures, 1.7 million of the forearm, and 1.4 million clinical vertebral fractures [1]. Of all these fractures, 51% are attributed to Europe and the Americas, while the rest belong to the Western Pacific region and Southeast Asia [1,2]. It is expected that in 30 years, the incidence of osteoporosis-related hip fracture will rise by 310% among men and 240% among women [3].

Bone tissue in osteoporosis changes in terms of mineral composition and bone density compared to healthy bones [4]. The decrease in bone density is called osteopenia and it is caused by aging-associated bone resorption, decline in the number of osteoblasts and reduced calcium absorption. A number of studies have been performed on developing strategies aiming to preserve bone mass and decrease the risks of the fractures. However, fewer efforts have been made to explore osteoporosis-associated fracture-healing strategies [5].

A number of promising approaches have been reported for the improvement of bone defects, including classical autologous and allogenic bone grafts, as well as novel strategies such as the application of growth factors and synthetic scaffolds [6,7,8,9,10]. However, osteoporotic fractures in aged patients are difficult to treat due to impaired healing and a problem of clinical fixation of the fracture in a weakened bone [11]. In recent years, there has been increasing evidence of affected bone healing processes in osteoporotic patients, which may play a crucial role in the assessment of new treatment strategies [5]. Thus, the development of alternative, clinically applicable therapies for the consequences of osteoporosis-associated and other pathological bone fractures is one of the priority areas of research.

There are several treatment strategies for osteoporosis, and the most common drugs used are bisphosphonates [12,13,14]. Bisphosphonates are chemical analogues of pyrophosphates (H_2_O_3_P–O–PO_3_H_2_), where the central group is a hydrolytically labile P–O–P linkage that has been substituted by the hydrolysis-resistant P–C–P bond. They selectively interact with hydroxyapatite groups at the site of bone resorption. Bisphosphonates also inhibit the enzyme farnesyl pyrophosphatase, which is of key importance in the metabolism of osteoclasts, by disrupting the functional activity of the osteoclasts and therefore stimulating bone formation. Bisphosphonate-based treatment is widely used in clinical practice to manage not only osteoporosis but also related conditions such as Paget’s disease. Bisphosphonates are also known to suppress the osteolytic activity of the cancer cells in bone [12].

Decrease in bone density and strength in osteoporosis happens not only due to increased osteoclastic activity, but also due to the age-related reduction in the number of osteoblast progenitors (i.e., mesenchymal stem cells, or MSCs). From this point of view, one of the most attractive approaches for the treatment of osteoporotic fractures is MSC therapy [15,16,17]. A method of treating osteoporosis is described where a patient is administered an intravenous biotransplant containing from 50 to 500 million MSCs [18]. Another approach is the implantation of cultured autologous or allogenic mesenchymal pluripotent stem cells injected into the bone injury zone to improve the processes of reparative osteogenesis [19,20,21]. The disadvantages of these methods are the lack of MSC’s affinity to bone tissue and the absence of the inhibition of the osteoclasts activity.

**Figure 1:**
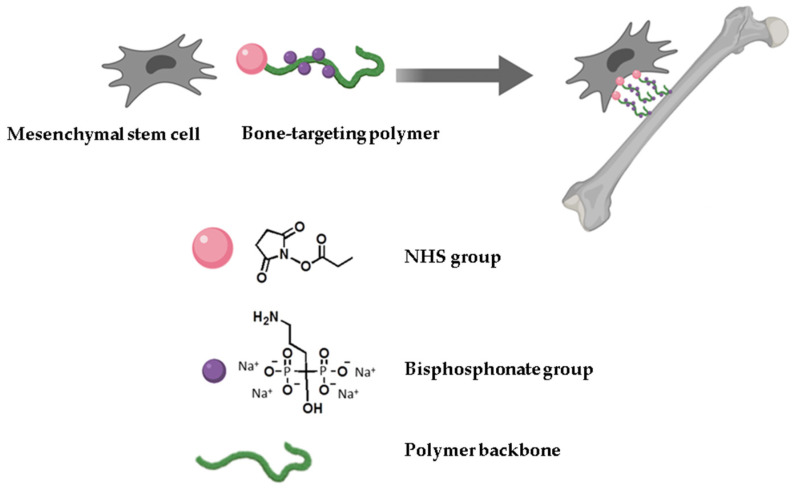
Schematic polymer structure with its functional groups.

We previously described a water-soluble polymer modified with bisphosphonate side chains that binds to MSCs and increases their affinity to bone in vitro [22]. Bisphosphonates selectively interact with the hydroxyapatites and disrupt the functional activity of the osteoclasts [12]. In addition to the bisphosphonates, the polymer contains N-succinimidyl-carboxylates (NHS) that react with cell surface amino groups to create covalently coupled polymer–cell complexes. The attachment of the polymer to MSCs allows the cells to specifically bind to the hydroxyapatite component of the bone (Figure 1). In the present study, we assessed the effects of the polymer on the MSCs’ proliferation, subsequent differentiation down to the osteogenic lineage, and the activity of the osteoclasts in vitro. We have also tested a local transplantation of MSCs that are coated with synthetic bisphosphonate-containing polymer for their ability to stimulate ulnar fracture regeneration in female rats with estrogen-dependent osteoporosis.

## 2. Materials and Methods

### 2.1. Polymer Synthesis

The polymer was synthesized using an atom transfer radical polymerization (ATRP), according to our previously published protocol [22]. Briefly, the core molecule, copolymer between dimethylacrylamide (DMAA), and acrylic N-hydroxysuccinimide (NHS) monomers, is modified by covalent binding to bisphosphonate following polymerization. ATRP works by adding successive monomers to one end of a growing polymer chain which results in the majority of the polymer chains undergoing near synchronous growth. For the cell binding moiety, the end modification of the polymer is also an NHS group giving the polymer the ability to bind to amino and carboxyl groups on the cell surface membranes. The synthesized polymers were characterized by gel permeation chromatography measures of molecular size (length), and NMR to determine the concentration of bisphosphonates.

### 2.2. Isolation of Rat MSCs

**Figure 2:**
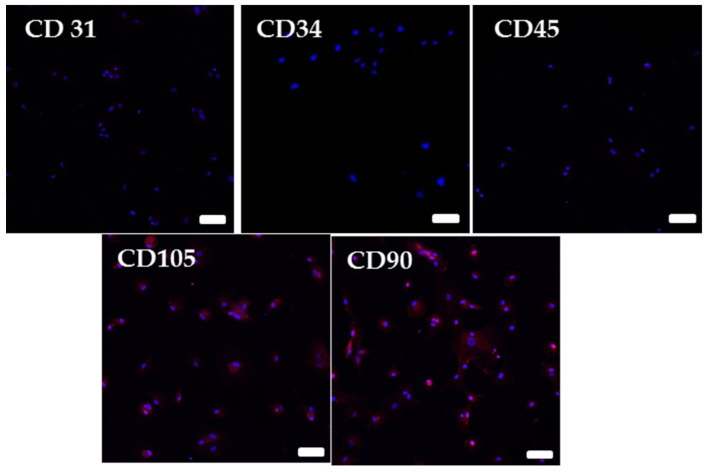
Immunofluorescent staining of the mesenchymal stem cells (MSCs) for the markers CD31, CD34, CD45, CD90 and CD105 with DAPI-stained nuclei; (calibration = 50 µm).

Bone marrow cells for our experiments were obtained from necroscopy samples of Wistar rats that were not treated with any compounds. MSCs were isolated according to a previously described procedure [23]. Briefly, femurs were harvested in sterile conditions, rinsed in a mixture of phosphate buffered saline (PBS) and antibiotics for 5 min, dissected of all soft tissue, transected at their epiphysis, and their marrow cavities were rinsed repeatedly with a mixture of heparin and Dulbecco’s minimum essential medium (DMEM, Gibco). The harvested cells were collected, centrifuged at 1000 rpm for 10 min. Cell pellets were resuspended with DMEM, and equal-volume percoll separator liquid with a density of 1.082 g/mL was added to a tube. The single nucleated cell layer was separated after centrifugation at 2000 rpm for 30 min. The MSC layer was resuspended in DMEM and centrifuged at 2000 rpm for 10 min. After washing, the cells were plated in DMEM culture medium that contained 10% fetal bovine serum (FBS) and cultured to the third passage. Cells were characterized as MSCs by flow cytometry. The mesenchymal nature of the sorted cells was confirmed by CD90, CD105, CD34, CD45 and CD31 immunofluorescent staining (Figure 2).

### 2.3. Transfection of MSCs with a Luciferase Gene Reporter Vector LVT-Luc2

Firefly luciferase lentiviral particles (LVT-Luc2) were used (Eurogen). Cells were seeded with final density 2 × 10^5^ per well in a 6-well plate. Lentiviral particles were thawed at room temperature. Two hundred microliters of lentiviral particles (0.5 × 10^6^ transduction units per mL) were added to each well. To enhance transfection, protamine sulfate (Sigma Aldrich, St. Louis, MO, USA) was used. Protamine sulfate was dissolved in MilliQ water and added to each well to obtain a final concentration of 100 µg/mL. The plate was incubated for 24 h at 37 °C, 5% CO_2_. After 24 h, the medium was changed to complete the DMEM (15% FBS, 1% Pen/Strep). Seventy-two hours later, the cells were assessed for transfection efficiency using the IVIS Spectrum CT (In Vivo Imaging Spectrum, Caliper, USA). For the in vitro bioluminescent assay, D-luciferin Firefly (Caliper, USA) was used. Stock solution was prepared at a concentration of 30 mg/mL in sterile water by gentle inversion, aliquoted and stored at −20 °C. Working solution was prepared in pre-warmed complete medium with the final concentration 150 µg/mL (1:200). Prior to imaging, the old medium was aspirated from the wells and Luciferin working solution was added to each well. The plate was assessed under IVIS in bioluminescence mode.

### 2.4. Coating of MSCs with the Polymer

LVT-Luc2-transfected MSCs (10^6^ cells) were incubated with the polymer (1 mg/mL in PBS, pH 8.0) for 10 min at 37 °C. After incubation, the cells were centrifuged at 300 g for 5 min, washed three times in PBS at pH 7.4.

### 2.5. Cell Viability Assay

Cell-Titer Glo Luminescent Cell Viability Assay (Promega, Madison, WI, USA) was used to assess the effect of the polymer on the MSCs’ proliferation in vitro. The number of metabolically active cells was quantified based of the ATP presence. MSCs (1.0 × 10^6^/^mL^) were incubated with 1 mg/mL of polymer for 10 min, washed 3 times with PBS, plated on a 96-well plate (Costar, Washington, DC, USA) and cultured for 0, 1, 2, 4, 24, 48 and 72 h at 37 °C, under 5% CO_2_. At certain time points, the CellTiter-Glo^®^ Reagent was added directly to the wells and shook for 2 min using an orbital shaker. To stabilize the luminescent signal, the plate was incubated at room temperature for 10 min. Luminescence signals were measured with a Biotek Hybrid Reader (Biotek, Winooski, VT, USA).

### 2.6. Osteogenic Differentiation of MSCs

Membrane-engineered MSCs were seeded in 24-well culture plates and incubated in complete DMEM (15% FBS, 1% Pen/Strep) for 12 h. Then, the medium was changed to an osteogenic medium (StemPro Osteogenesis Differentiation Kit, Invitrogen, Waltham, MA, USA) and the cells were cultured for 14 days. Osteogenic medium was changed every 2–3 days. As controls, non-modified and modified MSCs were cultured in complete DMEM. After 14 days of incubation, all the cells were stained for alkaline phosphatase (ALP).

### 2.7. Osteoclast Differentiation

Osteoclasts were derived from rat bone marrow according to the protocol of Tevlin et al. [24]. Briefly, bone marrow cells were isolated and separated using a gradient cell separation medium. Furthermore, additional cells were cultured in macrophage induction medium (MEM, 10% FCS, 1% Pen/Strep, 10 ng/mL macrophage colony-stimulating factor (M-CSF) and after 3 days changed to osteoclast induction medium (MEM, 10% FCS, 1% Pen/Strep, 10 ng/mL M-CSF + 10 ng/mL RANKL). The osteoclastic nature of the obtained cells was confirmed by staining with tartrate-resistant acid phosphatase (TRAP, Sigma Aldrich).

### 2.8. Bone Resorption Assay 

An osteoclast resorption assay was performed using a commercially available 24-well plate pre-coated with inorganic bone mimetic surface (Corning, Sigma). The cells were seeded at a concentration of 2 × 10^4^ per well. The next day, the medium was changed to contain the polymer at a concentration of 0.5, 1 and 2 mg/mL. The control group was treated with the normal osteoclast induction medium, and the commercially available alendronate (Landromax, GlobalPharm) was used as a positive control. On the 7th day of in vitro culture, the cells were incubated with the 10% bleach solution and counterstained with Von Kossa staining to visualize unresorbed substrate. Images were taken using Zeiss Microscope and analyzed using FIJI software for the resorbed and unresorbed area.

### 2.9. Animal Models of Osteoporosis and Ulnar Fracture 

Forty-five female Wistar rats at 12 weeks of age, with average weight between 200 and 300 g, were used in this study. The rats were kept in cages with a temperature of (22 ± 2) °C, a relative humidity of (55 ± 10)%, and a 12 h light/dark cycle (7:00 a.m. to 7:00 p.m.) with access to water and food ad libitum. All the experiments were executed according to the ethical guidelines of the U.S. Department of Health and Human Services (HHS), Registration of an Institutional Review Board (IRB) and agreed by the Ethics Committee of the Center for Life Sciences of Nazarbayev University (Registration number IORG 0006963).

Osteoporosis was induced in 40 females through bilateral ovariectomy (OVX) [25]. Later, three animals were excluded from the experiments due to unrelated health conditions. Five healthy female rats of the same age served as controls. Bone density was assessed before OVX and 3 months post-surgery using the microCT IVIS Spectrum (Caliper, USA). After confirming osteoporosis, we next created a non-critical ulnar defect. Under general anesthesia with isoflurane, standardized osteotomy was performed in left ulna shaft region 2.0 cm proximal to the radiocarpal joint. The ulnar was exposed by a 1.0 cm incision. The osteotomy was created using Liston bone-cutting forceps of 14.0 cm. The wound was washed with 0.9% NaCl solution and closed in layers. Following the operation, oral anesthetics were administered for 5 days to minimize animal distress.

Starting the next day after surgery, solutions were administered locally into the ulnar fracture zones (into the site of surgical incision) once per week for four weeks as follows: sham control (200 µL of PBS) as a negative control; 200 µL of PBS containing polymer alone (1 mg/mL); 1 × 10^6^ MSCs in 200 µL of PBS; and 1 × 10^6^ membrane-engineered MSCs in 200 µL of PBS. Bone density was measured locally at the zone of fracture the next day after surgery and over the next 4 and 24 weeks. Rats that were not subjected to OVX but with ulna fracture served as a positive control.

### 2.10. Survival Assessment of Transplanted MSCs

An evaluation of the viability and distribution of the luciferase-labeled cells at the fracture site was performed once per week with microCT (IVIS Spectrum CT, Caliper, USA). First, a fresh stock solution of Luciferin at 15 mg/mL was prepared in DPBS w/o Mg^2+^ and Ca^2+^ and sterilized through a 0.2 µm filter. Luciferin was injected at a concentration of 10 µL/g (150 mg/g) of body weight intra-peritoneally 10–15 min before imaging. A µCT machine (IVIS Spectrum CT, Caliper, USA) was used in Bioluminescence Mode. Images were obtained using the Living Image 4.3.1 software (Caliper, USA).

### 2.11. µCT Morphometry

A µCT machine (IVIS Spectrum CT; Caliper) was used in x-ray mode with 150 µ voxel size, 440 Al fitter, 50 kV, resolution 425, FOV L × W × H 12 × 12 × 13 cm. The approximate dose was 52 mGv per scan. The 3-D reconstruction and bone density assessments were performed using the Living Image 4.3.1 software (Caliper). The acquired image was exported in the Digital Imaging and Communications in Medicine (DICOM) format and stored. The density of the bone was defined as the optical density in the bone volume. A region of interest (ROI) was measured with a 10 mm cylindrical volume of interest positioned and the center of the fracture region.

### 2.12. Histological Assessment

The rats were sacrificed 4 and 24 weeks after the fracture by cervical dislocation under isoflurane anesthesia and the affected ulnar regions were removed. For the microscopic evaluation of regenerative processes at the fracture healing zone, the ulna was placed in 10% buffered formalin solution (pH 7.2–7.4). Bone fragments were decalcified, washed and embedded in paraffin. Microtome sections 7–10 microns in thickness were made from paraffin blocks, followed by hematoxylin and eosin staining.

### 2.13. Statistical Analysis

If data have passed the normality test, then they are presented as the mean ± SD and the mean differences between the experimental groups are tested using one-way ANOVA and/or unpaired t-test. If the data have failed the normality test, then they are presented as the median (IQR 0.25–0.75) and the Mann–Whitney Rank Sum Test is used to reveal differences between the experimental groups. Values are considered significantly different at the *p* ≤ 0.05 level. Statistical analyses were performed on the SigmaPlot 11.0 software.

## 3. Results

### 3.1. Effect of the Polymer on Viability and Osteogenic Differentiation of MSCs

**Figure 3:**
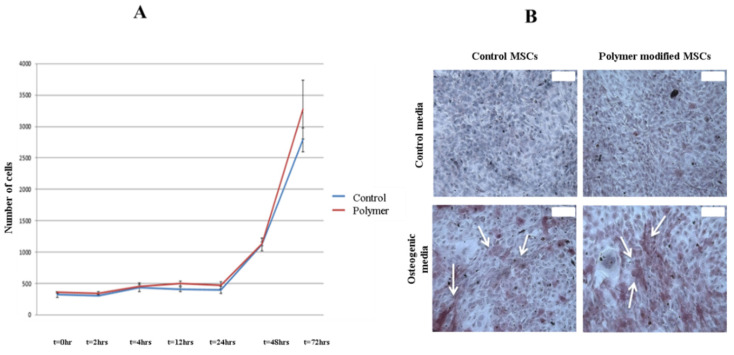
Effect of the polymer on the MSCs’ viability and osteogenic differentiation. (**A**) Cell viability assay (*p* = 0.383, one-way ANOVA); (**B**) the differentiation was performed for 3 weeks with Osteo Pro Differentiation Kit and assessed for early osteogenic activity with alkaline phosphatase (ALP) assay. Dense pink color is a marker for osteogenesis (areas shown with white arrows), 10× (calibration = 100 µm).

The results of the viability assay of MSCs coated with polymer and control (uncoated) cells are presented in Figure 3A. Consistently with our previous study, there was no significant difference between the viability of the control group and the membrane-engineered cells. Thus, the membrane engineering of rat MSCs with the polymer at a concentration of 1 mg/mL did not affect either the short-term (4 h) or long-term (72 h) cell growth in vitro. Difference between the groups was not statistically significant, *p* = 0.383.

The alkaline phosphatase staining of coated and uncoated MSCs incubated in PBS or osteogenic medium is presented on Figure 3B. Alkaline phosphatase is a marker of early osteogenesis and in the areas of increased alkaline phosphatase activity, a dark pink color is produced (white arrows). As seen from the images, there is no alkaline phosphatase activity in MSCs cultured in DMEM. Conversely, when cultured in osteogenic medium, both control MSCs and membrane-engineered MSCs expressed a similar level of alkaline phosphatase and these results are in agreement with our previous study [22].

### 3.2. Synthetic Polymer Inhibits Osteoclastic Activity In Vitro

**Figure 4:**
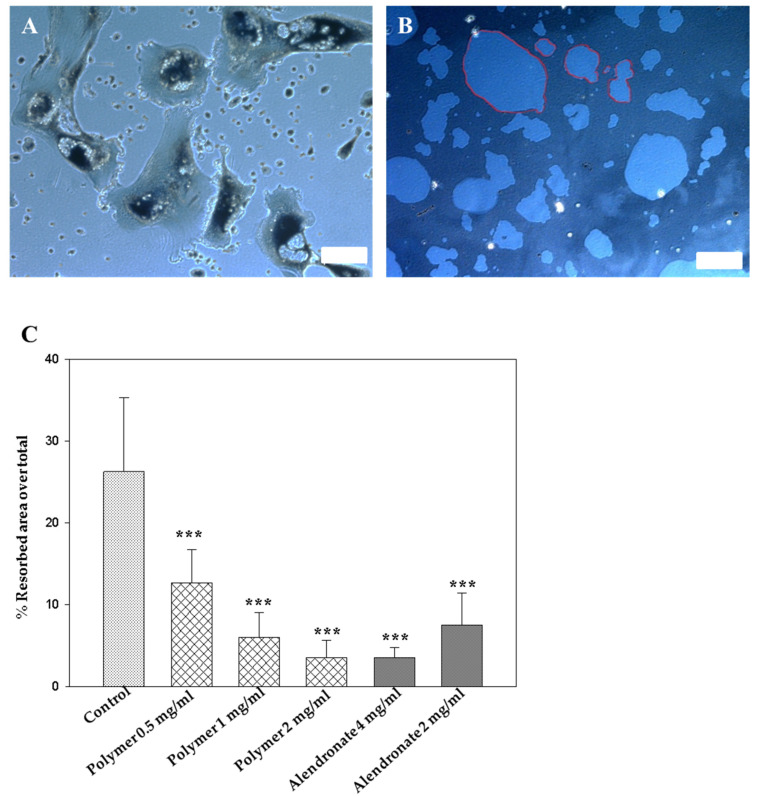
Synthetic polymer inhibits osteoclastic activity. (**A**) tartrate-resistant acid phosphatase (TRAP) staining of the osteoclasts, 10× (calibration = 100 µm); (**B**) pit assay: light areas—resorbed by osteoclasts, 10× (calibration = 100 µm); (**C**) quantitative analysis of pit assay: data are presented as a percentage of the resorbed area over the total area (data are presented as the mean ± SD, *** *p* ≤ 0.001, one-way ANOVA multiple comparisons with control group (Holm–Sidak method)).

Osteoclast differentiation of bone marrow-derived cells was confirmed by tartrate-resistant acid phosphatase (TRAP) staining. The high activity of TRAP is one of the key features of macrophages and osteoclasts. Osteoclasts are derived from the hematopoietic lineage that determines their similarity with macrophages. Another characteristic feature of osteoclasts is the presence of a large number of nuclei (from 10–40 to 100). Figure 4A shows a representative microphotograph of the osteoclast culture where the cells have a large number of nuclei; the shape of the cells is an irregular oval or polygonal, which sometimes has processes that gradually merge with the general background. Due to the functional features of osteoclasts (bone degradation), cells contain lysosomal vesicles.

To assess the effect of the polymer on the activity of osteoclasts, a pit assay was performed. As a reference substance, a commercially available alendronate (Londromax©, GlobalPharm) was used. Figure 4B shows images where lighter spots (indicated by arrows) are the bone-mimicking substance resorbed by macrophages. Resorbed area images were taken using the Zeiss Inverted Microscope Axio Observer and analyzed using FIJI software by calculating an un-resorbed area over the total area (Figure 4C). From the presented data, it is seen that in the control group the percentage of the resorption area was 26%. The polymer at a concentration of 0.5 mg/mL reduced the phagocytic activity of macrophages by 50% and by almost 85% at a concentration of 2 mg/mL, which was similar to the effect of alendronate at a concentration of 4 mg/mL. Thus, the results of the quantitative analysis show that the attachment to the polymer did not affect the osteoclast inhibitory properties of the bisphosphonate molecules.

### 3.3. Osteoporosis Modeling and In Vivo Optical Imaging of the Fractured Bones

**Figure 5:**
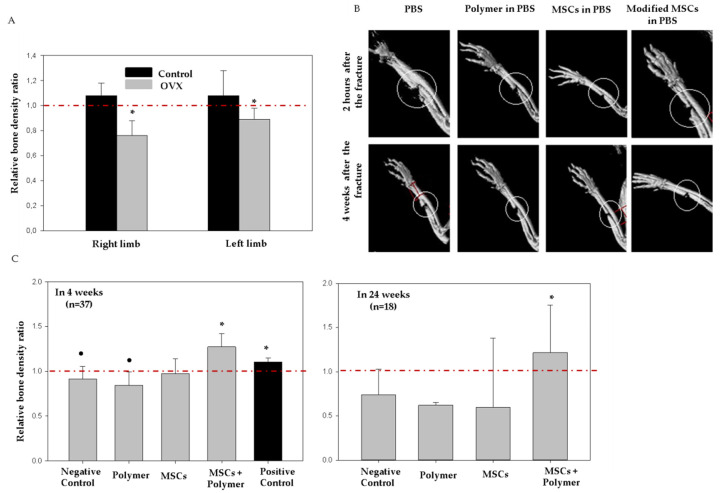
Bone density measurements and representative micro-CT images of the ulna fracture. (**A**) Osteoporosis rat model: bone density analysis in 5 control (no OVX) and 20 OVX animals; data are presented as the ratio of the final bone density (3 months) to the initial (before surgery); data are presented as the mean ± SD, * *p* ≤ 0.05 compared to the control (unpaired *t*-test); (**B**) the representative microCT images of the rat extremities after 2 h and 4 weeks post-surgery (In Vivo Imaging System, Caliper, USA); and (**C**) the bone density in the regions of the ulna fractures: data are presented as a proportion of final bone density (4 or 24 weeks after surgery) to the initial (2 h after surgery); the data are presented as the median (IQR 0.25–0.75), * *p* ≤ 0.05 compared to a negative control, • *p* ≤ 0.05—compared to a positive control (pairwise comparison using Mann–Whitney Rank Sum Test). Bone density was measured using microFCT IVIS Spectrum.

A model of estrogen-dependent osteoporosis in laboratory rats was created by OVX [25]. Ulnar fracture model was created in our lab based on the rabbit ulnar osteotomy model [26]. The model was used for the bone healing research and showed a decrease in bone mineral density under osteoporotic condition. The ulnar fracture was also convenient in a methodological way as no external fixation of the bone was needed. The adjacent radius serves as a splint and provides the weight-bearing support. Bone density was assessed one day before OVX and three months after surgery using the micro-CT IVIS Spectrum. We determined the ratios of final bone density (3 months after OVX) to initial pre-operation measurements in each animal. At a ratio equal to “1”, the bone density considered being unchanged; the ratio below “1” indicated decreased bone density. The induction of osteoporosis was considered successful if the bone density decreased by 10% or more, compared to the initial measurements. Figure 5A shows that in control animals the bone density slightly increased (by 8%), which was associated with the normal physiological maturation of the animals. In contrast, in the group of animals subjected to OVX, bone density decreased by ~20%, which indicated the development of estrogen-dependent osteoporosis.

After confirming osteoporosis, we then created open fractures in the ulnar regions. The following day we administered PBS, polymer, intact MSCs, and the membrane-engineered cells to the fracture zones. Bone density was evaluated at the fracture sites in 2 h. following the surgery and on 4th and 24th week. Differences in the bone density were calculated as a ratio of final bone density to initial density (at the time of fracture) in every individual rat. Then, the mean values were estimated for each group (Table 1). If the ratio was equal to 1, then the bone density did not change; values >1 were considered as indicators of increased bone density and bone regeneration.

As seen in Figure 5C and Table 1, four weeks after the surgery, there was slight decrease in bone density in the control group (by 8.6%), in the groups that received only polymer (15.5%) and only MSCs (2.4%), yet we did not observe statistically significant differences among those three groups. In contrast, in the group of animals injected with membrane-engineered MSCs, the bone density increased by 27.4% compared to the control (PBS). After 4 weeks, 19 animals were sacrificed and 18 animals were left for further observations. After 24 weeks, we repeated the measurements and found continuing decreases in bone density in groups 1–3 (26.3%, 37.9% and 40.4%, respectively), and there were still no statistical differences among these three groups. At the same time, in the fourth group, we observed a 21.5% increase in bone density compared to the negative control.

As a positive control, we used a group of animals with ulna fracture that were not subjected to OVX surgery (n = 5, positive control). As shown in Figure 5C and Table 1, there was a significant difference in bone density in this group in four weeks after the surgery compared to the negative control (OVX and ulna fracture). Since the healthy bones in rats regain their biomechanical properties by 4 weeks [27], we terminated the measurements at this point and used the same data as a positive control for further experiments.

Figure 5B shows the representative X-ray images of the fracture sites 2 h after the surgically performed fractures and 4 weeks later.

**Figure 6:**
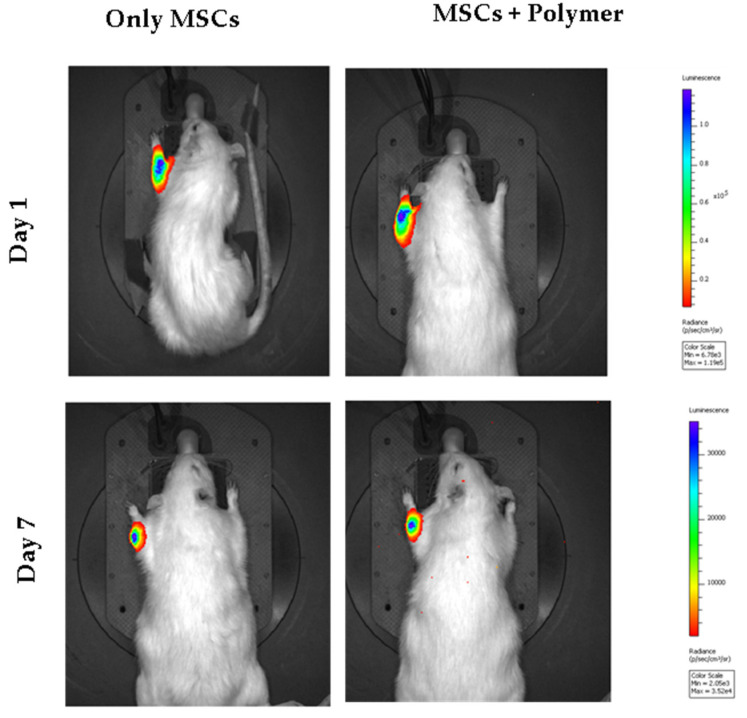
Bioluminescent analysis of the transplanted cells of the group that received MSCs only and polymer-coated MSCs. MSCs (1.0 × 10^6^ cells/mL) were transfected with firefly luciferase lentiviral particles (LVT-Luc2). Before analysis, luciferin was injected at a dose of 10 µL/g. Bioluminescence analysis was performed after 20 min.

To monitor the dynamics of the interstitial distribution of the transplanted cells, the MSCs were transfected with Luc-LVT lentiviral particles. To detect the bioluminescent signal of luciferin, an in vivo optical imaging system IVIS Spectrum CT was used. The results of a bioluminescent analysis for the luciferase expression are shown in Figure 6. The luminescent signal was clearly detected within one week after cell transplantation in MSCs and MSCs + polymer group, and we did not see any significant difference in the luminescent signal in those two groups. However, the signal could not be detected by the end of the second week in both groups. This could be either because of a low number of survived MSCs or the migration of the transplanted cell deep into the bone tissue, which makes it difficult to detect a bioluminescent signal due to the limited resolution of the imager.

### 3.4. Post Mortem Histological Assessment of the Regenerative Potential of MSCs Modified with Bone-Targeting Polymer

**Figure 7:**
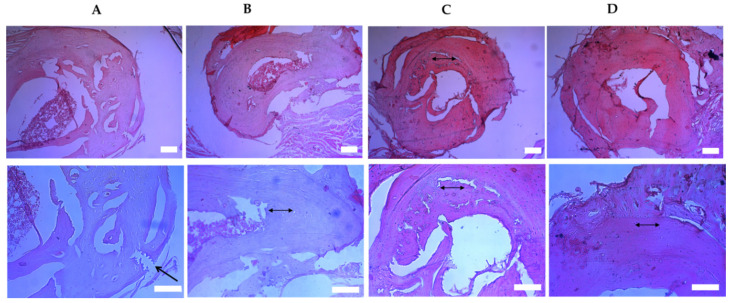
Histological changes within the fracture zones of ulna (cross-section) at 4 weeks after fracture (hematoxylin and eosin (H&E) staining; upper panel—10×, lower panel—20×). (**A**) PBS (negative control) group, showing fracture gaps, broken periosteum, endosteum; the broken end is clearly separated (an arrow), (calibration = 100 µm); (**B**) polymer group, showing immature bone tissue with fibrous tissue which started to close the fracture gaps (two-headed arrow), (calibration = 100 µm); (**C**) unmodified MSC group, showing bone regeneration and most of the bone tissue was connected to filling the gap (two-headed arrow), (calibration = 100 µm); (**D**) modified MSCs with a polymer group, showing the most prominent osteogenesis and the fracture gaps filled with bone tissue (two-headed arrow), (calibration = 100 µm).

Figure 7 represents the histological changes within the fracture zones of ulna (cross-section) at 4 weeks after fracture. Prominent signs of bone damage have been observed in the control group in four weeks after fracturing. In particular, the fracture gaps, broken periosteum, and endosteum could be seen. In the second group of animals, the polymer alone caused a slight beneficial effect, since we observed immature bone tissue with fibrous tissue started to close the fracture gaps. In the group injected with unmodified MSCs, the bone tissue revealed some signs of regeneration. In particular, we were able to see bone tissue connecting to filling the gap. However, the most noticeable osteogenesis was observed in the group injected with membrane-engineered MSCs, where the fracture gaps were filled with bone tissue. These observations are consistent with bone density measurements in ulna fracture zones after 4 weeks since the treatment, where we observed a 27.4% increase in the bone density in the group of animals injected with membrane-engineered MSCs compared to the negative control.

**Figure 8:**
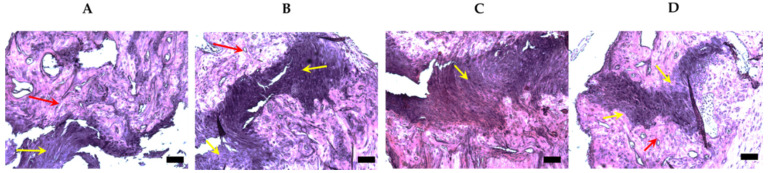
Longitudinal sections of ulna within fracture zones in 24 weeks upon fracturing (H&E staining; 10X). (**A**) PBS (negative control) group, showing multiple bone defects crossing the shear axis of the bones were still present. Bone tissue adjacent to the fracture showed moderate dystrophic changes (red arrow). Immature cartilaginous tissues (yellow arrow) filled in the fracture gaps (calibration = 100 µm); (**B**) polymer groups, showing uneven growths of immature cartilaginous tissues without clear boundaries in the fracture gaps (yellow arrows). There were also moderate to severe dystrophic changes in bone tissue (red arrow) (calibration = 100 µm); (**C**) unmodified MSC groups, showing outgrowth of the immature cartilaginous tissue (yellow arrow) and a large number of polymorphic chondroblasts chaotically distributed within cartilaginous tissue (calibration = 100 µm); and (**D**) modified MSCs with the polymer group, showing prominent osteogenesis and ossification (yellow arrows). Fracture gaps start to be filled with mature bone tissue. No pronounced dystrophic and degenerative changes in the bone sections (red arrow) (calibration = 100 µm).

Histological slices of ulna within fracture zones by 24 weeks upon fracturing are presented on Figure 8. As it could be seen from the images, in the PBS control group, multiple bone defects crossing the shear axis of the bones were still present. Bone tissue adjacent to the fracture showed moderate dystrophic changes and immature cartilaginous tissues filled in the fracture gaps could be seen. In the second group (polymer in PBS), uneven growths of immature cartilaginous tissues without clear boundaries in the fracture gaps were observed. There were also moderate to severe dystrophic changes in bone tissue.

On the histological slices of the third group (injected with unmodified MSCs) were visible fragments of bone tissue with moderate dystrophic changes, including where the outgrowth of the immature cartilaginous tissue and a large number of polymorphic chondroblasts were chaotically distributed within the cartilaginous tissue. At the same time, we did not observe the pronounced dystrophic and degenerative changes in the bone sections from the fourth group of animals. Bone tissue demonstrated the sights of osteogenesis and ossification. Fracture gaps started to be filled with mature bone tissue.

Thus, consistently with the micro-CT data, the histological assessment of ulnar fracture zones demonstrated a significant difference in the reactive and reparative processes across the experimental groups, with the most prominent regenerative outcomes derived from the treatment with polymer-modified MSCs.

## 4. Discussion

Delayed fracture healing in estrogen-dependent osteoporosis is associated with an increase in osteoclast activity and a decreased number of MSCs [28,29]. In this regard, the transplantation of MSCs is a promising approach for the treatment of bone pathologies especially in age-associated conditions such as osteoporosis [21,30]. However, the isolation of a clinically relevant number of autologous MSCs from aged individuals is problematic and the cells must be expanded in vitro. In turn, the in vitro expansion of MSCs alters its surface receptors’ profile and affects its homing ability compared to that of freshly isolated MSCs [31,32,33]. In addition, transplanted MSCs tend to home to the sites of abnormal cell proliferation such as breast cancer [34]. In order to overcome these limitations, we added bone-targeting moiety to MSCs through membrane engineering with bisphosphonate-containing polymers that have a high affinity for hydroxyapatite. The coating of MSCs with the polymer allowed the cells to bind specifically to the HA component of bone [22]. The in vitro polymer was shown to be non-cytotoxic while not interfering with the differentiating potential of the MSC. Besides having a targeting moiety, bisphosphonates also inhibit farnesyl pyrosphosphate synthase (FPPS), a key enzyme in osteoclast metabolism. Alendronate groups in the polymer preserved its functional activity compared to the therapeutic doses of commercially available alendronate analogues.

For the in vivo assessment of the fracture-regenerating potential of MSCs coated with bone-targeting polymer in osteoporotic conditions, we created a model of estrogen-dependent osteoporosis by OVX. There are different animal models that mimic post-menopausal estrogen-dependent type I osteoporosis and age-related type II osteoporosis. As most of the osteoporotic conditions correspond to the estrogen-dependent type, the OVX animal model is generally accepted and approved by the FDA [35].

We also developed a model of non-union bone fracture created by ulnar osteotomy. The non-union bone fracture in healthy rats are expected to regain its biomechanical properties by 4 weeks [27] and heal completely by 12 weeks [36,37]. However, osteoporotic condition delays the fracture repair. According to the studies of Namkung-Matthai et al. osteoporosis affects fracture healing in the early stage and results in a 23% decrease in bone density and reduced bone callus formation after 3 weeks of fracture [38]. Data acquired by Kubo et al. showed impaired bone regeneration in the late period of fracture healing. By 12 weeks radiological and histological analysis revealed decreased bone density and diminished callus quantity that impaired woven bone formation [39]. In agreement with published studies, our results demonstrated that bone density in the control group declined by 8.6% in 4 weeks and dropped further to 26.3% in 24 weeks.

The group of animals that received only the bisphosphonate polymer showed a similar 15.5% decrease in bone density after 4 weeks which, however, proceeded to a dramatic 37.9% decline after 24 weeks. However, these data are not statistically significant and need further investigation. In turn, the injection of plain MSCs resulted in a mild decrease in bone density after 4 weeks, but by 24 weeks, the bone density reached the same 40.4% decrease as in the polymer group. These data might indicate that MSCs had some beneficial effect immediately after their injection but does not have a prolonged effect. In contrast, the group that received coated MSCs showed pronounced statistically significant increase in bone density (27.4%) in the fracture zone after 4 weeks. The long-term effect of the injection of polymer-modified MSCs led to the persistent maintenance of bone density at 21.5% monitored after 24 weeks, although the histological picture did not significantly improve compared to 4 weeks. It might be a consequence of ongoing osteoporotic process since we discontinued treatment after the fourth week.

Fracture healing generally proceeded in three phases. The first phase stage was reactive in nature and was characterized by fracture hematoma, inflammation, and the formation of granulation tissue. During the second reparative phase, collagen fibers connected the broken bone ends, while osteoblasts started to form spongy bone. Some spicule may also appear at this point. The resulting fibrocartilaginous callus is converted into rigid calcified tissue (woven bone) by endochondral ossification. The final phase is bone remodeling, during which the bone is restored to its original shape, structure, and mechanical strength.

In our study, the histological assessment of the control group after 4 weeks demonstrated some evidence of the early reparative processes with the fibrous union of old bone fragments. Groups 2 and 3 had additional evidence of mineralization and bone spicule formation, while the group with polymer-coated MSCs showed active osteogenesis with the formation of a fibrocartilaginous callus. Although we did not observe complete fracture healing by 24 weeks in any of the groups, the most pronounced formation of woven bone was seen in group 4. We suggested that surface modification with bisphosphonate groups enhanced the regeneration process in two ways: first, by increasing the recruitment of transplanted MSCs to bone damage sites, thus providing growth factors and potential differentiation into the osteoblast cell, and second by disrupting the functional activity of the osteoclasts and therefore decreasing the process of bone resorption.

## 5. Conclusions

In conclusion, in the present study, we demonstrated that the use of MSCs coated with a synthetic bone-targeted bisphosphonate polymer was a more effective method for stimulating reparative osteogenesis in the zone of the delayed fusion of osteoporosis-associated fractures compared to the treatment with unmodified MSCs. Therefore, this combined approach that involves polymer and cell transplantation shows promise and warrants further biosafety and clinical exploration.

## Figures and Tables

**Table 1:** Ratios of the bone densities measured at 4th and 24th week after the fracture to the initial bone densities (in two hours after surgery).

Animal Group	Relative Bone Density Ratio (after Fracture/before Fracture) Median (iqr 0.25–0.75)	
	4 Weeks	24 Weeks
Negative control (OVX, ulna fracture)	0.914 (IQR 0.671–1.053) (*n* = 9)	0.737 (IQR 0.640–1.029) (*n* = 4)
Polymer	0.845 (IQR 0.727–0.994) (*n* = 10) * *p* = 1.00 • *p* = 0.032	0.621 (IQR 0.435–0.652) (*n* = 5) * *p* = 0.066 • *p* = 0.008
MSCs	0.976 (IQR 0.674–1.141) (*n* = 8) * *p* = 0.597 • *p* = 0.354	0.596 (IQR 0.453–1.379) (n = 4) * *p* = 0.486 • *p* = 0.268
MSCs + polymer	1.274 (IQR 1.046–1.421) (*n* = 10) * *p* = 0.003 • *p* = 0.058	1.215 (IQR 1.124–1.754) (n = 5) * *p* = 0.032 • *p* = 0.095
Positive control (no OVX, ulna fracture)	1.103 (IQR 0.971–1.148) (*n* = 5) * *p* = 0.046	No data

Note: Mann–Whitney Rank Sum Test was used to compare the differences between the experimental groups: *—compared to a negative control; •—compared to a positive control. OVX—ovariectomy.

## References

[1] Johnell O., Kanis J.A. (2006). An estimate of the worldwide prevalence and disability associated with osteoporotic fractures. Osteoporos. Int. A J. Establ. Result Coop. Between Eur. Found. Osteoporos. Natl. Osteoporos. Found. USA.

[2] Hernlund E., Svedbom A., Ivergard M., Compston J., Cooper C., Stenmark J., McCloskey E.V., Jonsson B., Kanis J.A. (2013). Osteoporosis in the European Union: Medical management, epidemiology and economic burden. A report prepared in collaboration with the International Osteoporosis Foundation (IOF) and the European Federation of Pharmaceutical Industry Associations (EFPIA). Arch. Osteoporos..

[3] Gullberg B., Johnell O., Kanis J.A. (1997). World-wide projections for hip fracture. Osteoporos. Int..

[4] Chen L., Yang L., Yao M., Cui X.J., Xue C.C., Wang Y.J., Shu B. (2016). Biomechanical Characteristics of Osteoporotic Fracture Healing in Ovariectomized Rats: A Systematic Review. PLoS ONE.

[5] Giannoudis P., Tzioupis C., Almalki T., Buckley R. (2007). Fracture healing in osteoporotic fractures: Is it really different? A basic science perspective. Injury.

[6] Nakase T., Fujii M., Myoui A., Tamai N., Hayaishi Y., Ueda T., Hamada M., Kawai H., Yoshikawa H. (2009). Use of hydroxyapatite ceramics for treatment of nonunited osseous defect after open fracture of lower limbs. Arch. Orthop. Trauma Surg..

[7] James R., Deng M., Laurencin C.T., Kumbar S.G. (2011). Nanocomposites and bone regeneration. Front. Mater. Sci..

[8] Kamrani R.S., Mehrpour S.R., Sorbi R., Aghamirsalim M., Farhadi L. (2013). Treatment of nonunion of the forearm bones with posterior interosseous bone flap. J. Orthop. Sci..

[9] Jeon O.H., Elisseeff J. (2016). Orthopedic tissue regeneration: Cells, scaffolds, and small molecules. Drug Deliv. Transl. Res..

[10] An S.H., Matsumoto T., Miyajima H., Nakahira A., Kim K.H., Imazato S. (2012). Porous zirconia/hydroxyapatite scaffolds for bone reconstruction. Dent. Mater..

[11] Pesce V., Speciale D., Sammarco G., Patella S., Spinarelli A., Patella V. (2009). Surgical approach to bone healing in osteoporosis. Clin. Cases Miner. Bone Metab..

[12] Bone H.G., Hosking D., Devogelaer J.P., Tucci J.R., Emkey R.D., Tonino R.P., Rodriguez-Portales J.A., Downs R.W., Gupta J., Santora A.C. (2004). Ten years’ experience with alendronate for osteoporosis in postmenopausal women. N. Engl. J. Med..

[13] Cranney A., Wells G., Willan A., Griffith L., Zytaruk N., Robinson V., Black D., Adachi J., Shea B., Tugwell P. (2002). Meta-analyses of therapies for postmenopausal osteoporosis. II. Meta-analysis of alendronate for the treatment of postmenopausal women. Endocr. Rev..

[14] Wells G., Cranney A., Peterson J., Boucher M., Shea B., Robinson V., Coyle D., Tugwell P. (2008). Risedronate for the primary and secondary prevention of osteoporotic fractures in postmenopausal women. Cochrane Database Syst. Rev..

[15] Cho S.W., Sun H.J., Yang J.Y., Jung J.Y., An J.H., Cho H.Y., Choi H.J., Kim S.W., Kim S.Y., Kim D. (2009). Transplantation of mesenchymal stem cells overexpressing RANK-Fc or CXCR4 prevents bone loss in ovariectomized mice. Mol. Therapy.

[16] Teitelbaum S.L. (2010). Stem cells and osteoporosis therapy. Cell Stem Cell.

[17] Yao W., Lane N.E. (2015). Targeted delivery of mesenchymal stem cells to the bone. Bone.

[18] Yamada Y., Boo J.S., Ozawa R., Nagasaka T., Okazaki Y., Hata K., Ueda M. (2003). Bone regeneration following injection of mesenchymal stem cells and fibrin glue with a biodegradable scaffold. J. Craniomaxillofac. Surg..

[19] Singh J., Onimowo J.O., Khan W.S. (2014). Bone marrow derived stem cells in trauma and orthopaedics: A review of the current trend. Curr. Stem Cell Res..

[20] Tasso R., Ulivi V., Reverberi D., Lo S.C., Descalzi F., Cancedda R. (2013). In vivo implanted bone marrow-derived mesenchymal stem cells trigger a cascade of cellular events leading to the formation of an ectopic bone regenerative niche. Stem Cells Dev..

[21] Huang S., Xu L., Zhang Y., Sun Y., Li G. (2015). Systemic and Local Administration of Allogeneic Bone Marrow-Derived Mesenchymal Stem Cells Promotes Fracture Healing in Rats. Cell Transpl..

[22] D’Souza S., Murata H., Jose M.V., Askarova S., Yantsen Y., Andersen J.D., Edington C.D., Clafshenkel W.P., Koepsel R.R., Russell A.J. (2014). Engineering of cell membranes with a bisphosphonate-containing polymer using ATRP synthesis for bone targeting. Biomaterials.

[23] Zhang L., Chan C. (2010). Isolation and enrichment of rat mesenchymal stem cells (MSCs) and separation of single-colony derived MSCs. J. Vis. Exp..

[24] Tevlin R., McArdle A., Chan C.K., Pluvinage J., Walmsley G.G., Wearda T., Marecic O., Hu M.S., Paik K.J., Senarath-Yapa K. (2014). Osteoclast derivation from mouse bone marrow. J. Vis. Exp. JoVE.

[25] Mathavan N., Turunen M.J., Tagil M., Isaksson H. (2015). Characterising bone material composition and structure in the ovariectomized (OVX) rat model of osteoporosis. Calcif. Tissue Int..

[26] Waters R.V., Gamradt S.C., Asnis P., Vickery B.H., Avnur Z., Hill E., Bostrom M. (2000). Systemic corticosteroids inhibit bone healing in a rabbit ulnar osteotomy model. Acta Orthop. Scand..

[27] Funk J.R., Hale J.E., Carmines D., Gooch H.L., Hurwitz S.R. (2000). Biomechanical evaluation of early fracture healing in normal and diabetic rats. J. Orthop. Res. Off. Publ. Orthop. Res. Soc..

[28] Cheung W.H., Miclau T., Chow S.K., Yang F.F., Alt V. (2016). Fracture healing in osteoporotic bone. Injury.

[29] Yousefzadeh N., Kashfi K., Jeddi S., Ghasemi A. (2020). Ovariectomized rat model of osteoporosis: A practical guide. Excli J..

[30] Kiernan J., Hu S., Grynpas M.D., Davies J.E., Stanford W.L. (2016). Systemic Mesenchymal Stromal Cell Transplantation Prevents Functional Bone Loss in a Mouse Model of Age-Related Osteoporosis. Stem Cells Transl. Med..

[31] Rombouts W.J., Ploemacher R.E. (2003). Primary murine MSC show highly efficient homing to the bone marrow but lose homing ability following culture. Leukemia.

[32] Sackstein R., Merzaban J.S., Cain D.W., Dagia N.M., Spencer J.A., Lin C.P., Wohlgemuth R. (2008). Ex vivo glycan engineering of CD44 programs human multipotent mesenchymal stromal cell trafficking to bone. Nat. Med..

[33] Leibacher J., Henschler R. (2016). Biodistribution, migration and homing of systemically applied mesenchymal stem/stromal cells. Stem Cell Res. Ther..

[34] Kidd S., Spaeth E., Dembinski J.L., Dietrich M., Watson K., Klopp A., Battula V.L., Weil M., Andreeff M., Marini F.C. (2009). Direct evidence of mesenchymal stem cell tropism for tumor and wounding microenvironments using in vivo bioluminescent imaging. Stem Cells.

[35] McCann R.M., Colleary G., Geddis C., Clarke S.A., Jordan G.R., Dickson G.R., Marsh D. (2008). Effect of osteoporosis on bone mineral density and fracture repair in a rat femoral fracture model. J. Orthop. Res. Off. Publ. Orthop. Res. Soc..

[36] Grundnes O., Reikeras O. (1992). Blood flow and mechanical properties of healing bone. Femoral osteotomies studied in rats. Acta Orthop. Scand..

[37] Mills L.A., Simpson A.H. (2012). In vivo models of bone repair. J. Bone Jt. Surgery. Br. Vol..

[38] Namkung-Matthai H., Appleyard R., Jansen J., Hao Lin J., Maastricht S., Swain M., Mason R.S., Murrell G.A., Diwan A.D., Diamond T. (2001). Osteoporosis influences the early period of fracture healing in a rat osteoporotic model. Bone.

[39] Kubo T., Shiga T., Hashimoto J., Yoshioka M., Honjo H., Urabe M., Kitajima I., Semba I., Hirasawa Y. (1999). Osteoporosis influences the late period of fracture healing in a rat model prepared by ovariectomy and low calcium diet. J. Steroid Biochem. Mol. Biol..

